# An Endoscopic View of Multiple Hemangiomas of the Small Intestine

**DOI:** 10.7759/cureus.44221

**Published:** 2023-08-27

**Authors:** Medha Reddy, Donelle Cummings

**Affiliations:** 1 School of Medicine, New York Medical College, Valhalla, USA; 2 Advanced Endoscopy/Gastroenterology, New York Medical College at Metropolitan Hospital Center, New York, USA

**Keywords:** abdominal bleeding, unexplained abdominal pain, chronic abdominal pain, small bowel hemangioma, small intestine neoplasm

## Abstract

Small intestinal hemangiomas may present as a severe gastrointestinal hemorrhage associated with a hematologic emergency. In the emergent setting, this may result in more extensive intestinal resection than would otherwise be necessary with elective intervention. The widespread application of capsule endoscopy and double-balloon enteroscopy presents an opportunity to diagnose small bowel hemangiomas prior to symptomatic onset. In one of the first published cases of multiple small intestinal hemangiomas, we highlight the importance of maintaining a broad differential and pursuing a thorough workup, including small bowel imaging, in patients with complaints of chronic abdominal pain and anemia.

## Introduction

Small bowel hemangiomas, a rare form of gastrointestinal neoplasms, have a preoperative diagnosis rate of less than 30% [1,2]. With the rise of capsule endoscopy and double-balloon enteroscopy imaging modalities, clinicians have an opportunity to address this staggering low preoperative diagnosis rate and to improve their patient’s clinical outcomes significantly. Here, we present one of the first preoperatively diagnosed, published cases of multiple small intestinal hemangiomas.

## Case presentation

A 25-year-old male with a history of uncontrolled hypertension, gastroesophageal reflux disease, and fractures of the spine and pelvis secondary to mechanical trauma over five years ago, status post-fixation of the symphysis pubis and bilateral sacroiliac joints, presented for an outpatient gastrointestinal consultation following an emergency department encounter for a six-year history of epigastric abdominal pain. He described the epigastric pain as intermittent, sharp, and progressively worsening. The patient complained of abdominal “fullness,” not associated with his intermittent bouts of constipation. Upon presentation, he denied vomiting, diarrhea, blood in stool, melena, weight loss, change in appetite, recent travel, and sick contacts.

**Figure 1:**
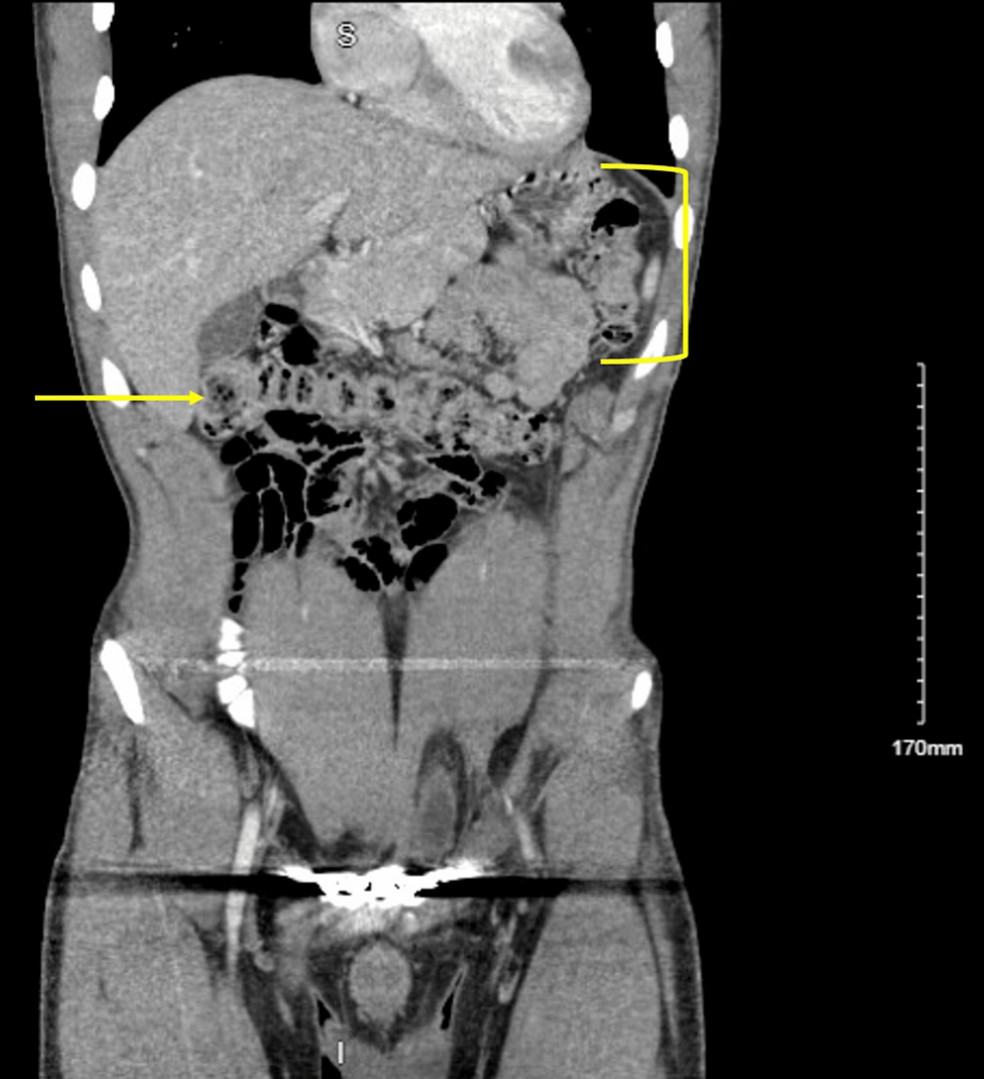
CT abdomen and pelvis demonstrating the relationship between the transverse colon (arrow) and one of the regions dense in filling defects, the ileum (bracket).

**Figure 2:**
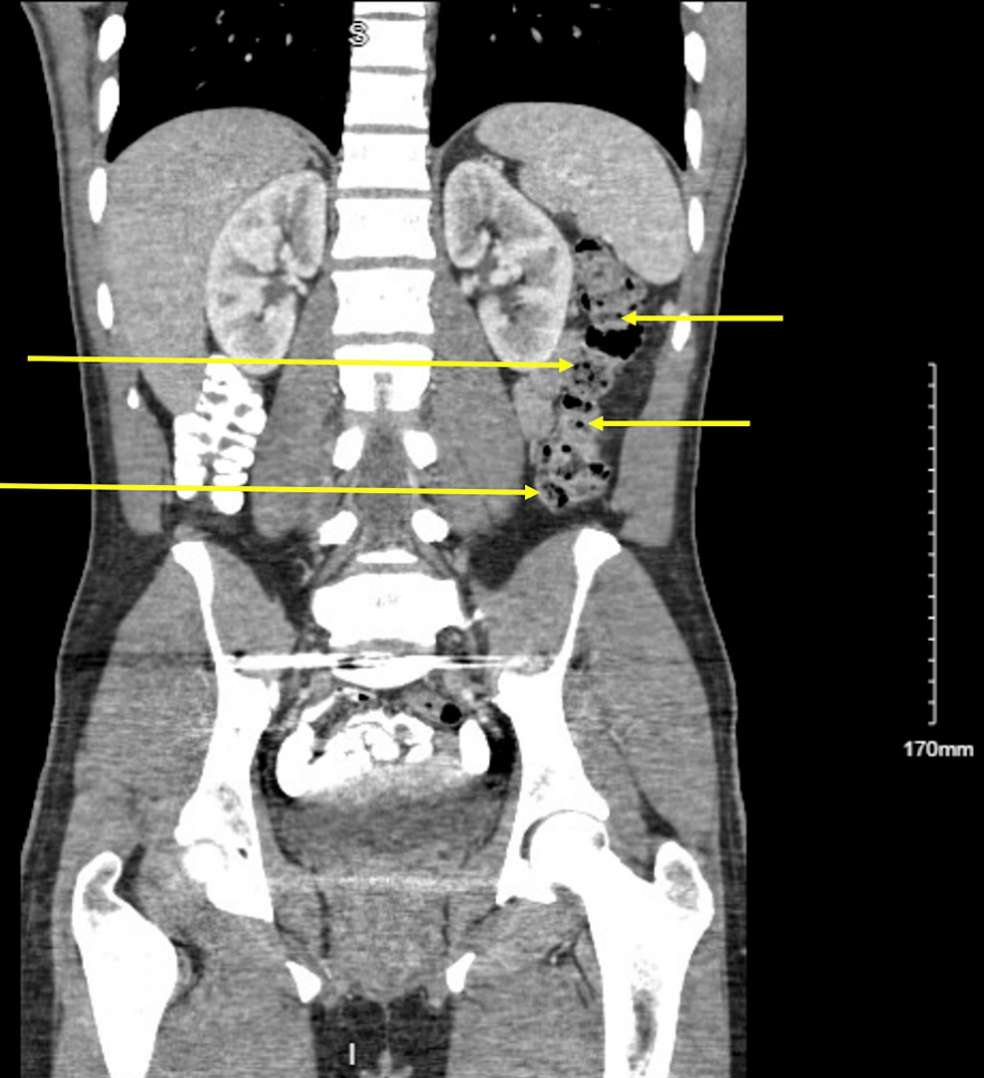
CT abdomen and pelvis demonstrating several filling defects throughout the ileum (arrows).

**Figure 3:**
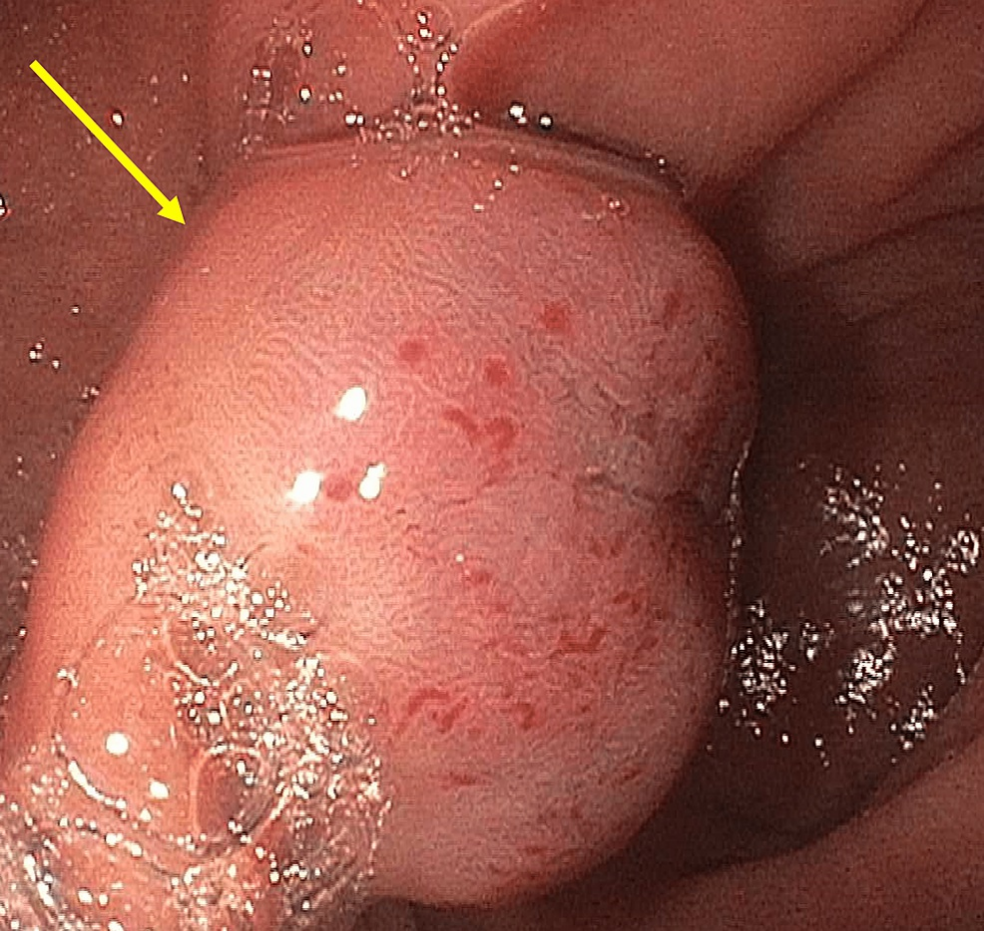
Esophagogastroduodenoscopy (EGD) demonstrating a friable vascular mass without bleeding in the duodenal bulb, with biopsy suggestive of hemangioma.

On initial examination, lower right abdominal quadrant tenderness to palpation was appreciated. Complete blood count was notable for microcytic anemia (hemoglobin (Hg) 8.6, hematocrit (Hct) 30.2, mean corpuscular volume (MCV) 64.8, mean corpuscular hemoglobin concentration (MCHC) 28.5, mean corpuscular hemoglobin (MCH) 18.5, and red cell distribution width (RDW) 17.2). Computed tomography (CT) abdomen and pelvis demonstrated a 10-mm-diameter soft tissue density filling defect in the ileum, presumed to be a colonic polyp (Figures 1, 2). Esophagogastroduodenoscopy (EGD) demonstrated large 2.5 cm friable vascular-appearing polyploid mass with no bleeding found in the duodenal bulb (Figure 3). Biopsy of the lesion showed few dilated vascular spaces in mucosa, suggestive of a hemangioma, and Brunner glands identified in the mucosa. Colonoscopy showed no evidence of colonic abnormalities.

## Discussion

**Figure 4:**
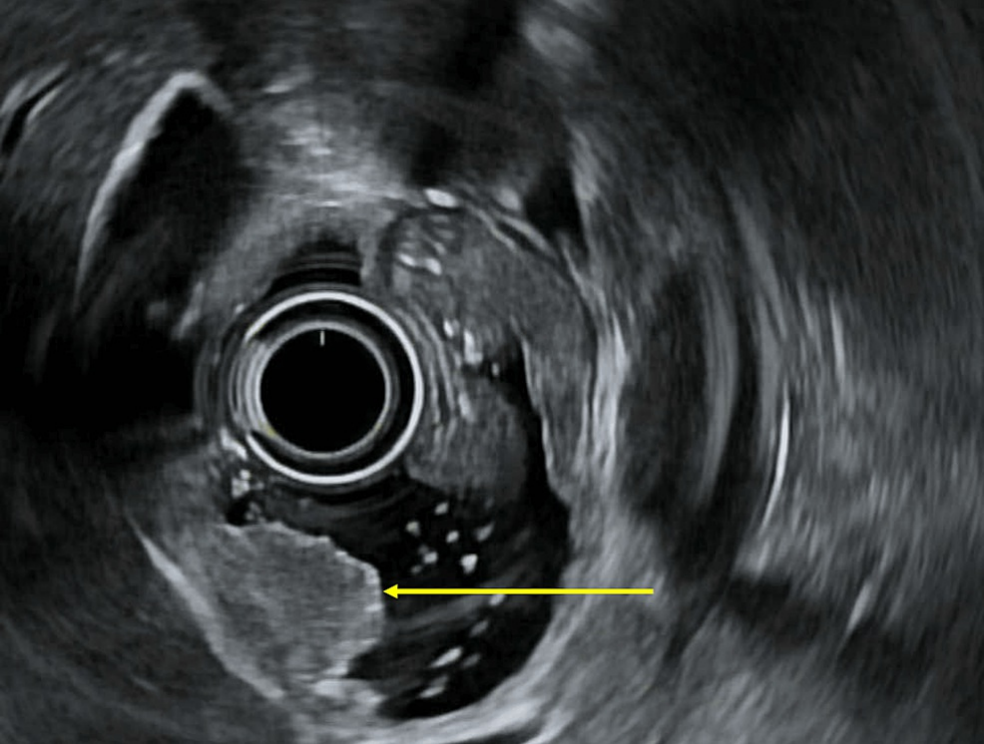
Endoscopic ultrasound demonstrating a 15 mm lesion in the deep mucosa.

**Figure 5:**
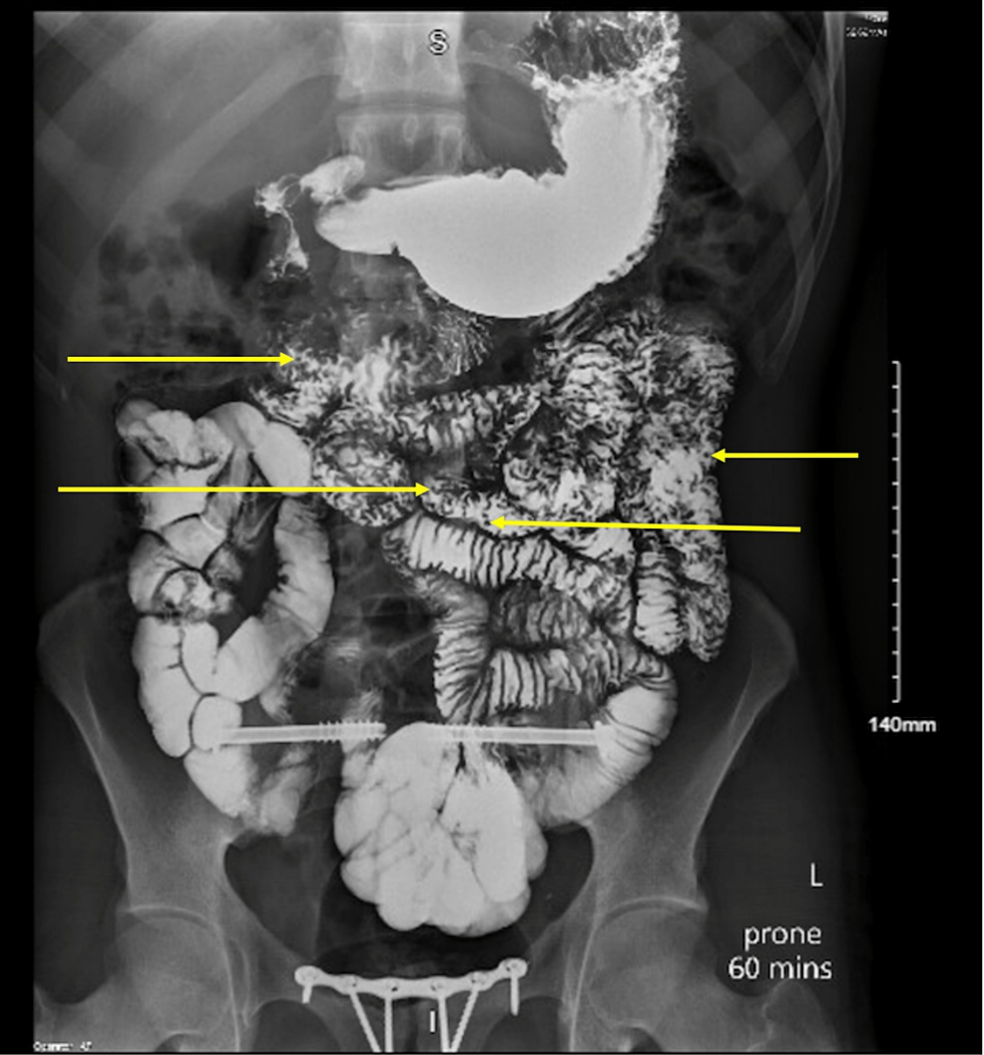
Upper gastrointestinal series with small bowel follow-through showing multiple luminal filling defects in the distal jejunum and ileum.

The patient was started on a senna-based bowel regimen, which provided minimal relief. Follow-up endoscopic ultrasound revealed a subepithelial lesion in the duodenal bulb, which appeared to originate from the superficial mucosal-luminal interface (Figure 4). Subsequent fluoroscopic small bowel follow-through demonstrated multiple filling defects in the distal jejunal and ileal lumen (Figure 5). Further endoscopy and surgical resection were deferred due to the high risk of the hemangioma's bleeding and the goals of care established by the patient. 

The patient has been started on a bowel regimen and is undergoing watchful waiting. As of today, the patient’s symptoms persist. He has been referred to a small bowel surgical specialist for consultation.

Hemangiomas are symptomatic in 90% of cases, typically presenting as anemia of unknown origin secondary to chronic gastrointestinal bleeding [3]. A lack of timely diagnosis and intervention by endoscopists and surgeons may lead to emergent gastrointestinal bleeding and more extensive intestinal resection than would be necessary with elective endoscopic intervention or laparoscopic excision [1]. Physicians must consider the application of capsule endoscopy and double-balloon enteroscopy imaging modalities in order to ensure a comprehensive workup of possible small bowel pathologic disorders in patients with a history of chronic abdominal pain and unidentified sources of gastrointestinal bleeding.

While resection of the affected segment of bowel has become the mainstay of treatment, few have weighed the curative effect of widespread intestinal resection against the drastic consequences upon patient quality of life, which proved to be a critical factor in the decision-making of our patient [3-5]. Although some have utilized polypectomy, sclerotherapy, and cauterization for lesions accessible via endoscopy, these approaches largely remain underutilized and understudied given the friability of these structures and the risk of uncontrollable hemorrhage [3,6-7]. Although medical therapies, such as corticosteroids and interferon-gamma, have proved promising in hemangioma treatment during childhood, little work has been done to evaluate their utility among adults with hemangiomas [3,8]. The overall scarcity of literature on the topic indicates a need for further study to elucidate the optimal means of treating these patients following diagnosis.

## Conclusions

In one of the first published cases of multiple small intestinal hemangiomas that were preoperatively diagnosed, we highlight how the increased availability of new imaging modalities can improve patient outcomes by allowing clinicians to plan more conservative surgical interventions outside of emergent settings. Our patient's diffuse distribution of small intestinal hemangiomas, as well as the surgical challenge posed by balancing the friability of the structures with the consequences of intestinal resection, underscores the need for further surgical literature on this topic. 

In patients presenting with abdominal pain and microcytic anemia, but without overt gastrointestinal bleeding, it is critical that physicians maintain a broad differential that includes pathologic disorders of the small intestine and utilize adjunctive diagnostic techniques, including endoscopy and small bowel imaging, to reach a conclusive diagnosis. 
